# Antimalarial evaluation of alkyl-linked bis-thiadiazine derivatives in murine model infected with two *Plasmodium* strains

**DOI:** 10.5599/admet.2105

**Published:** 2023-11-30

**Authors:** Katherine Loachamin-Gualotuña, Lilian M. Spencer, Hortensia Rodriguez, Abigail Montero-Calderon, Beatriz Pernia, Julieta Coro, Margarita Suarez, Francisco Javier Tingo-Jácome, Zully J. Rodriguez-Parra, José Manuel Lozano, Jesús A. Cortés-Vecino

**Affiliations:** 1 School of Biological Sciences and Engineering, University of Investigation and Experimental Technology Yachay, 100650, Ecuador; 2 Simón Bolívar University, Valle de Sartenejas, Cell Biology Department, Venezuela; 3 School of Chemical Sciences and Engineering, University of Investigation and Experimental Technology Yachay, 100650, Ecuador; 4 School of Agricultural and Agro-industrial Sciences, University of Investigation and Experimental Technology Yachay, 100650, Ecuador; 5 University of Guayaquil, Faculty of Natural Sciences, Guayaquil, Ecuador; 6 Laboratorio de Síntesis Orgánica, Facultad de Química, Universidad de La Habana, 10400, Cuba; 7 Biology Center, Central University of Ecuador, Ecuador; 8 Universidad Nacional de Colombia, Laboratorio de Parasitología Veterinaria, Grupo de Investigación Parasitología Veterinaria, Colombia; 9 Universidad Nacional de Colombia, Departamento de Farmacia, Mimetismo Molecular de los Agentes Infecciosos, Colombia; 10 Universidad Nacional de Colombia, Departamento de Salud Animal, Facultad de Medicina Veterinaria y Zootecnia, Colombia

**Keywords:** *Plasmodium berghei*, *Plasmodium yoelii*, Bis-thiadiazines, drugs, parasitemia, humoral response

## Abstract

**Background and Purpose:**

*Plasmodium falciparum* and *P. vivax* are responsible for most malaria cases in humans in the African Region and the Americas; these parasites have developed resistance to classic antimalarial drugs. On the other hand, previous investigations of the alkyl-linked bis tetrahydro-(2H)-1,3,5-thiadiazine-2-thione (bis-THTT) derivatives compounds show satisfactory results against protozoan parasites such as *Trypanosoma cruzi*, *Trypanosoma vaginalis*, *Trypanosoma brucei rhodesiense* and *Leishmania donovani*. Therefore, it is possible to see some effect of bis-THTT derivatives on other protozoan parasites, such as *Plasmodium*.

**Experimental Approach:**

This study aimed to perform an *in vivo* biological evaluation of bis-THTT (JH1 to JH6) derivatives compounds as possible anti-malaria drugs in BALB/c mice infected with *Plasmodium berghei* ANKA and *Plasmodium yoelii* 17XL strains. In this work, we evaluated the compounds as potential antimalarial drugs in BALB/c mice infected with *Plasmodium* strains.

**Key Results:**

For each compound, we assess the percentages of parasitemia by smears from tail blood and the humoral response by indirect ELISA test using each compound as an antigen. We also evaluated the B lymphocyte response and the cytotoxicity of the bis-THTT derivatives compounds with MTT cell proliferation assays.

**Conclusions:**

Our results show that the bis-THTT derivatives JH2 and JH4 presented effective parasitemia control in mice infected with *P. berghei*; JH5 and JH6 compounds have similar infection control results as chloroquine in mice infected *P. yoelii* strain. The evaluation of bis-THTT derivatives compounds in a model of BALB/c mice infected with *P. berghei* and *P. yoelii* allowed us to conclude that some of them have an antimalarial effect; however, none of the tested compounds exceeded the efficiency of chloroquine.

## Introduction

Malaria is a global public health problem; this disease is one of the main causes of anemia in infants and pregnant women, low weight in newborns, premature births, and infant mortality [1]. In fact, of all malaria deaths in Africa, 80 % correspond to children under the age of five [2]. The genus *Plasmodium* causes malaria and has a large geographic distribution in 87 tropical and subtropical countries such as Africa, Asia, and America [1]. The high rate of transmission increases in endemic areas; thus, Brazil, Colombia, and Venezuela account for 77 % of all malaria cases in the Region of the Americas [2].

Malaria in humans is caused by five species of the genus *Plasmodium*, which are *P. ovale, P. malariae, P. knowlesi, P. falciparum* and *P. vivax*, of which the last two species are responsible for the majority of malaria cases at the Americas [1,2]. *Plasmodium berghei, P. chabaudi, P. vinckei* and *P. yoelii* are species of murine *Plasmodium*; minor differences exist in the biology of the rodent malaria parasites, and this makes them interesting models to investigate different aspects of human malaria [3,4].

On the other hand, malaria is an infectious disease that is preventable and treatable. Malaria treatment aims to achieve the complete cure, which is the rapid and total elimination of the *Plasmodium* parasite from the patient’s blood. Artemisinin-based combination therapy (ACT) is used as first-line therapy for uncomplicated infections with *P. falciparum* [1]. ACT combines two active ingredients with different action mechanisms; it consists of an artemisinin component (artesunate, artemether, or dihydroartemisinin), a rapidly reducing parasitemia, and a second partner antimalarial drug that slowly eliminates the residual parasites. The oral ACT treatments have few adverse effects. They are available as fixed-dose combinations (artemether-lumefantrine, dihydroartemisinin-piperaquine, artesunate-sulfadoxine / pyrimethamine artesunate-amodiaquine, artesunate-mefloquine, and recently added artesunate-pyronaridine) [5].

Similarly, chloroquine (CQ) has been used as an antimalarial drug for more than 70 years, so resistance developed slowly and progressively [6]. The plasmodial mechanism of CQ is the inhibition of heme polymerase activity. Usually, when *Plasmodium spp*. is in the merozoite or trophozoite stage, they are inside red blood cells. They degrade hemoglobin to acquire essential amino acids, which parasites require as protein sources and energy metabolism. The degradation of hemoglobin generates high amounts of the free heme group, whose oxidation releases excess electrons and favours the generation of reactive oxygen species. Likewise, the parasite polymerizes the heme group to form hemozoin (malaria pigment) [7]. CQ-sensitive parasites accumulate large amounts of chloroquine in their digestive vacuoles, unlike CQ-resistant parasites. Other studies with *P. falciparum* proposed that this difference may be due to a point mutation in the gene that codes for the protein PfCRT, found in the digestive vacuole membrane [8,9].

The *P. vivax* infections are treated with an ACT or CQ. In areas where *P. vivax* has been identified as chloroquine-resistant, infections should be treated with an ACT, preferably in which the partner medicine has a long half-life. Except for the artesunate + sulfadoxine-pyrimethamine (AS+SP) combination, all ACTs are effective against the blood-stage infections of *P. vivax*. Primaquine should be added to the treatment to avoid relapses; the dose and frequency of the administration should be guided by the patient’s glucose-6-phosphate dehydrogenase (G6PD) enzyme activity [1,10].

Nowadays, there is no effective vaccine against malaria, and the development of parasite resistance to drugs is one of the greatest threats to malaria control. Resistance to classical antimalarial medicines has been confirmed in two of the five human malaria parasite species: *P. falciparum*, and *P. vivax* [2]. *Plasmodium falciparum* has developed resistance to nearly all currently available antimalarial drugs, such as sulfadoxine/pyrimethamine, mefloquine, halofantrine, and quinine [11]. Therefore, it is necessary to research and evaluate new treatments to eradicate malaria disease.

Due to their lipophilicity, the tetrahydro-(2*H*)-1,3,5-thiadiazine-2-thione (THTT) have great potential in drug research as bio-labile prodrugs. THTT derivatives could pass through biological membranes to the required sites of action, thus improving their bioavailability [12].

In this regard, several *in vitro* and *in vivo* tests have shown that THTT derivatives have antibacterial, antifungal, anthelmintic, and tuberculostatic properties. For these excellent physicochemical properties, THTT derivatives have become a key molecular scaffold of an integral project for the development of new antiparasitic agents [13,14].

Following the interest in antiprotozoal drugs, the synthesis and biological evaluation of two new series of alkyl-linked bis-tetrahydro-(2*H*)-1,3,5-thiadiazine-2-thione (bis-THTT) derivatives were reported. In this synthetic approach, two THTT rings were incorporated into the same molecular structure to improve the antiprotozoal effect [13]. Alkyl-linked bis-(2-thioxo-[1,3,5]thiadiazinan-3-yl) carboxylic acids derivatives were evaluated for their activity *in vitro* against *Trypanosoma cruzi* strain CL (clone CL B5) and *Trichomonas vaginalis* strain JH 31A. The preliminary biological evaluation demonstrated that some compounds have notable activity against *T. cruzi* and *T. vaginalis* [13].

In a second study, other alkyl-linked bis-(2-thioxo-[1,3,5] thiadiazinan-3-yl) carboxylic acids show *in vitro* antiprotozoal activity against *Leishmania donovani, Trypanosoma brucei rhodesiense* STIB900, and *Plasmodium falciparum* 3D7 strains. The results were satisfactory only for *T. b. rhodesiense* [15].

In 2008, the synthesis and *in vitro* antiprotozoal evaluation of novel N4-(benzyl) spermidyl-linked bis-THTT derivatives from N4-(benzyl) spermidine were carried out. These new bis-THTTs were evaluated *in vitro*, against *L. donovani, T. cruzi, T. b. rhodesiense* STIB900, *and P. falciparum* 3D7 strain parasites. The preliminary results showed potent protozoocidal activity against *T. cruzi* and *L. donovani* [16].

**Figure 1.**
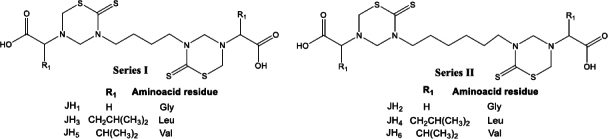
Alkyl-linked bis-thiadiazine structures. Series I: JH1, JH3, and JH5. Series II: JH2, JH4, and JH6.

Herein, we synthesized and evaluated for the first time the *in vivo* effect of six alkyl-linked bis-THTT belonging to series I and II (Figure 1) as antimalarial drugs in BALB/c mice infected with *Plasmodium berghei* ANKA and *Plasmodium yoelii* 17XL strains. Besides, we used these compounds as antigens to evaluate the humoral response in BALB/c mice by indirect ELISA test. Another aspect of this work was the stimulation of B lymphocytes by compounds tested with a cell proliferation assay. In addition, cytotoxicity was evaluated for each bis-THTT compound using the MTT colorimetric method with neuroblastoma cells; our results showed neither compound was cytotoxic.

## Experimental

### Chemistry

All starting materials were commercially available. All reagents were of analytical grade, dried, and purified when necessary. Melting points were determined in an Electrothermal apparatus and were not corrected. TLC analytical silica gel plates monitored the progress of the reaction and purity of compounds (Merck F254). Spectra were obtained as follows: FTIR was recorded on a Bruker IRS48 spectrometer; ^1^H NMR spectra were recorded at 400 MHz, and ^13^C NMR at 101 MHz, on a Bruker Avance-400 instrument.

### General procedure for {5-[(carboxyalkyl)-methyl]-2-thioxo-[1,3,5]thiadiazinan-3-yl]-alkyl}-6-thioxo-[1,3,5]-thiadiazinan-3-yl]-carboxylic acids (JH1-JH6)

To a stirred solution of the corresponding diamine (5 mmol) in 25 ml of water, potassium hydroxide (0.56 g, 10 mmol, as a 20 % aqueous solution) and carbon disulfide (0.6 ml, 10 mmol) was added at room temperature. The mixture was subsequently stirred for 4 h. Then, a formaldehyde solution 37 % (1.6 ml, 20 mmol) was added, and the stirring continued for 1 h. The reaction mixture was added dropwise to a suspension of the corresponding amino acid or glycyl-glycine (10 mmol) in a pH 7.8 buffer solution of Phosphate (10 ml), stirred for 2 h, and filtered off. The aqueous solution was cooled in an ice bath and acidified to pH 2 by 15 % hydrochloric acid. In most cases, the obtained precipitate was filtered and then kept in a vacuum drier overnight. The solid residue was crushed with cold ether and filtered.

*JH1: 5-[6-(5-Carboxymethyl-2-thioxo-[1,3,5]thiadiazinan-3-yl)-butyl]-6-thioxo1,3,5]thiadiazinan-3-yl}-acetic acid.* From 1,4-diaminobutane (5 mmol, 0.5 ml) and glycine (10 mmol, 0.75 g); yield (1.95 g, 88 %); melting point 134 to 136 °C. IR (KBr/cm) 2923 (*υ* OH); 2854 (*υ*_as_ CH_2_); 1737 (*υ* C=O); 1502 (*υ* C=S); 1425 (*δ* OH); 1329 (*υ* C-O + *υ* C-N); 903 (*γ* OH). NMR ^1^H (400 MHz, DMSO-d_6_) *δ*= 4.53 (m, 8H, 2×[CH_2_], 2×[CH_2_]); 3.93 (m, 4H, 2×(CH_2_]); 3.51 (s, 4H, 2×[CH_2_]); 1.58 (m, 4H, 2×[CH_2_]). NMR ^13^C (101 MHz, DMSO-d6) *δ* = 190.7; 171.1; 70.0; 58.5; 51.1, 51.2; 23.5. MS-ESI: [M-H]^-^ = 436.9 Calculated for C_14_H_22_N_4_O_4_S_4_: 437,59 g/mol.

*JH2: {5-[6-(5-Carboxymethyl-2-thioxo-[1,3,5]thiadiazinan-3-yl)-hexyl]-6-thioxo1,3,5]thiadiazinan-3-yl}-acetic acid.* From 1,6-diaminohexane (5 mmol, 0.7 ml) and glycine (10 mmol, 0.75 g); yield (2.18 g, 93 %); melting point 130 to132 °C. IR (KBr/cm) 3437 (*υ* NH); 2930 (*υ* OH); 2854 (*υ*_as_ CH_2_); 1738 (*υ* C=O); 1505 (*υ* C=S); 1422 (*δ* OH); 1329 (*υ* C-O + *υ* C-N); 897 (*γ* OH). NMR ^1^H (400 MHz, DMSO-d_6_) *δ*= 4.51 (s, 4H, 2×[CH_2_]); 4.50 (s, 4H, 2×[CH_2_]); 4.00-3.84 (m, 4H, 2×(CH_2_]); 3.50 (s, 4H, 2×[CH_2_]); 1.55 (m, 4H, 2×[CH2]); 1.26(m, 4H, 2×[CH2]). MS-ESI: [M-H]^-^ = 464.9 Calculated for C_16_H_26_N_4_O_4_S_4_: 465.65 g/mol.

*JH3: 2-(5-{6-[5-(1-Carboxy-3-methyl-butyl)-2-thioxo-[1,3,5]thiadiazinan-3-yl]-butyl}-6-thioxo-[1,3,5]thiadiazinan-3-yl)-4-methyl pentanoic acid.* From 1,4-diaminobutane (5 mmol, 0,5 ml) and leucine (10 mmol, 1.31 g); yield (0.63 g, 38 %); melting point 132–134 °C. IR (KBr/cm) 3076 (*υ* Csp2-H) + (*υ* COOH); 2928 (*υ* OH), 2871 (*υ*_as_ CH_2_), 1720 (*υ* C=O), 1494 (*υ* C=S), 1325 (*υ* C-O), 924 (*γ* O-H). NMR ^1^H (400 MHz, DMSO-d_6_) *δ*= 4.65 (d, *J* = 13.8 Hz, 4H, 2×[CH_2_]); 4.53 (d, *J* = 16.6 Hz, 4H, 2×[CH_2_]); 4.10-3.80 (m, 4H, 2×[CH_2_]); 3.60-3.45 (m, 2H, 2×[CH]); 1.75-1.45 (m, 10H, 2×[CH_2_]-8, 2×[CH_2_], 2× CH]; 0.90 (m, 12H, 4×[CH_3_]). NMR ^13^C (101 MHz, DMSO-d_6_) δ= 191.2; 173.7; 67.9; 61.2; 56.2; 49.4; 39.1; 25.3; 23.7; 22.8, 22.0, MS-ESI: [M-H]^-^ = 549.0 Calculated for C_22_H_38_N_4_O_4_S_4_: 549.81 g/mol.

*JH4: 2-(5-{6-[5-(1-Carboxy-3-methyl-butyl)-2-thioxo-[1,3,5]thiadiazinan-3-yl]-hexyl}-6-thioxo-[1,3,5]thiadiazinan-3-yl)-4-methyl pentanoic acid.* From 1,6-diaminohexane (5 mmol, 0.7 ml) and leucine (10 mmol, 1.31 g); yield (0.76 g, 57 %); melting point 95–97 °C. IR (KBr/cm) 3070 (*υ* Csp2-H) + (*υ* COOH); 2921 (*υ* OH); 2864 (*υ*_as_ CH_2_); 1705 (*υ* C=O); 1495 (*υ* C=S); 1331 (*υ* C-O) + *υ* C-N); 924 (*γ* OH). NMR ^1^H (400 MHz, DMSO-d_6_) *δ*= 4.64 (d, *J* = 13.8 Hz, 4H, 2×[CH_2_]); 4.53 (d, *J* = 14.9 Hz, 4H, 2×[CH_2_]); 4.02-3.74 (m, 4H, 2×[CH_2_]); 3.57-3.47 (m, 2H, 2×[CH]); 1.70-1.46 (m, 10H, 2×[CH_2_], 2×[CH_2_], 2×[CH]); 1.29 (m, 4H, 2×[CH_2_]); 0.95-0.85 (m, 12H, 4×[CH_3_]). NMR ^13^C (101 MHz, DMSO-d_6_) *δ*= 190.9; 173.8; 67.9; 61.; 56.1; 52.0; 39.0; 26.4; 25.3; 23.8, 22.3, MS-ESI: [M-H]^-^ = 577.0 Calculated for C_24_H_42_N_4_O_4_S_4_: 577.86 g/mol.

*JH5: 2-(5-{6-[5-(1-carboxy-1-metyl-propil)-2-thioxo-[1,3,5]-tiadiazinan-3-yl]-butyl}-6-thioxo-[1,3,5]tiadiazinan-3-yl)-3-methylbutanoic acid.* From 1,4-diaminobutane (5 mmol, 0.5 ml) and valine (10 mmol, 1.17 g); yield (0.72 g, 46 %); melting point 121–124 °C. IR (KBr/cm) 3090 (*υ* Csp2-H) + (*υ* COOH); 2922 (*υ* OH); 2863 (*υ*_as_ CH_2_); 1705 (*υ* C=O); 1495 (*υ* C=S); 1424 (*δ* OH); 1328 (*υ* C-O + *υ* C-N); 930 (*γ* OH); 671 (*γ* CH). NMR ^1^H (400 MHz, DMSO-d_6_) *δ*= 4.73-4.42 (m, 8H, 2×[CH_2_], 2×[CH_2_]); 4.02-3.85 (a, 4H, 2×[CH_2_]); 3.22 (d, 2H, *J* = 7.3 Hz, 2×[CH]); 2.17-2.01 (m, 2H, 2×[CH_2_]); 1.75-1.45 (m, 4H, 2×[CH_2_]); 0.95 (d, 6H, *J* = 6.6 Hz, 2×[CH_3_]); 0.89 (d, 2H, *J* = 6.6 Hz, 2×[CH_3_]). NMR ^13^C (101 MHz, DMSO-d_6_) *δ*= 191.0; 172.6; 69.8; 67.9; 56.4; 51.6; 27.9; 23.8; 20.1; 17.8, MS-ESI: [M-H]^-^ = 521.0 Calculated for C_20_H_34_N_4_O_4_S_4_: 521.76 g/mol.

*JH6: 2-(5-{6-[5-(1-carboxy-1-metyl-propil)-2-thioxo-[1,3,5]-tiadiazinan-3-yl]-hexyl}-6-thioxo-[1,3,5]tiadiazinan-3-yl)-3-methylbutanoic acid.* From 1,6-diaminohexane (5 mmol, 0.7 ml) and valine (10 mmol, 1.17 g); yield (0.86 g, 52 %); melting point 131–133 °C. NMR ^1^H (400 MHz, DMSOd_6_) *δ*= 4.67-4.42 (m, 8H, 2×[CH_2_]-4, 2×[CH_2_]-6); 3.95-3.80 (m, 4H, 2×[CH_2_]); 3.21 (d, *J* = 7.1 Hz, 2H, 2×[CH]); 2.15-2.00 (m, 2H, 2×[CH_2_]); 1.59 (a, 4H, 2×[CH_2_]); 1.27 (a, 4H, 2×[CH_2_]); 0.93 (d, 6H, *J* = 6.7 Hz, 2×[CH_3_]); 0.87 (d, 2H, *J* = 6.5 Hz, 2×[CH_3_]). MS-ESI: [M-H]^-^ = 549.0 Calculated for C_22_H_38_N_4_O_4_S_4_: 549.81 g/mol.

### Malaria parasite

Strains of *Plasmodium yoelii* 17XL and *Plasmodium berghei* ANKA were used in experimental *in vivo* assays. The parasites were kept as cryopreserved (20 % glycerol at -80 °C) and were activated by intraperitoneal (IP) inoculation of 200 μL of the cryopreserved into adult BALB/c mice of approximately 10 weeks old. For each parasite strain, the parasitemia was monitored daily by microscopic examination of blood smear stained with Giemsa’s reagent until a value equal to or greater than 40 % of parasitemia was reached.

### Obtaining parasitized red blood cells (PRBC) with P. berghei and P. yoelii to inoculate different groups of experimental mice

Mice previously inoculated and reached at least 40 % parasitemia were sacrificed, and blood was collected using heparinized Krebs solution (0.9 % NaCl + 4.2 % sorbitol in a 1:20 dilution of Heparin). The schizont and trophozoite stage parasites were isolated through a cellulose column (CF-11, Sigma) with a NaCl buffer 0.9 %. The white blood cells were retained in the cellulose column. The sample eluted from the cellulose column was centrifuged at 2000 rpm for 5 minutes at 4 °C. Subsequently, the supernatant was discarded, and the pellet was resuspended at twice the volume with the Krebs solution. The concentration of parasitized red blood cells was determined using a Neubauer chamber to adjust the concentration of 5×10^3^ PRBC/200μl for each inoculation.

### Experimental inoculation with P. berghei and P. yoelii to BALB/c mice

Once the concentration of PRBC was determined in the Neubauer chamber, the inoculum with parasites of *P. berghei* and *P. yoelii* was prepared so that in 200 μL of inoculum, there are 5×10^3^ PRBC. The inoculum was administered intraperitoneally to female mice BALB/c about 10 weeks old.

### Preparation and administration of treatments with bis-THTTs (JH1-JH6)

Six bis-tetrahydro-(2H)-1,3,5-thiadiazine-2-thione derivatives (JH1–JH6) were evaluated as antimalarial drugs, and the CQ was used as the positive control of cure assays. The chemical structure of bis-THTT consists of two THTT rings, connected via their N-3 atom by a linear aliphatic backbone and bearing carboxyl residues at N-5. Also, we used 7-chloro-N-[5-(diethylamino)pentan-2-yl]quinolin-4-amine (Chloroquine), which is a 4-aminoquinoline.

### Experimental treatment assay with compounds derived from bis-THTT to BALB/c mice infected with P. berghei and P. yoelii

The administration scheme of compounds derived from bis-THTT was carried out for four consecutive days (after 3 days of the infection with *Plasmodium* strain). The compounds were administered orally using a solution of methylcellulose 1 % and Tween 80 at 0.5 % as a dosing vehicle [17]. The dose used for the six compounds derived from bis-THTT and CQ was 20 mg/kg, calculated according to the average weight of the mice, in a final volume of 200 μL of treatment per mouse. To dissolve the experimental compounds derived from bis-THTT, we used dimethyl-sulfoxide (DMSO) for its solvent power and excellent ability to transport active principles [18]. The concentration of DMSO should not exceed 10 % of the total volume because it can be toxic to experimental animals. The Ethical Review Board approved all mice experimentation at Universidad Nacional de Colombia-Bogotá.

### Obtaining pre-immune and hyperimmune sera of each bis-THTT (JH1-JH6) and chloroquine

Eight groups of female BALB/c mice were used to obtain the sera: one group for each bis-THTT experimental compound, one for CQ, and another for the control group parasite infection; each experimental group had three mice. The tail end of each mouse was cut to obtain pre-immune sera, and blood was collected without anticoagulant, the obtained sera were stored at -20 °C until later use [19,20].

The obtaining of hyperimmune sera was carried out after immunizing the female BALB/c mice intraperitoneally, administering 200 μL of the experimental compound and Freund’s adjuvant. Immunizations were performed four times: the first immunization was performed with complete Freund’s adjuvant, while the remaining three immunizations were with incomplete Freund’s adjuvant. Five days after the last immunization, the mice were sacrificed, and the blood was collected. After the formation of the blood clot, the sample was centrifuged for 20 minutes at 2500 rpm, and the serum was extracted and stored at -20 °C [19,20].

### Indirect ELISA test using the experimental compounds as antigens

The protocol described by [21,22] was followed to obtain optimal conditions of the indirect ELISA test. ELISA plates (96-well Nunc) were sensitized, with 100 μL of the soluble compounds in each well at increasing concentrations of 5 to 10 μg/ml diluted in carbonate-bicarbonate buffer at pH 9.6 (0.5 M Na_2_CO_3_ and 0.35 M NaHCO_3_). The plates were incubated in a humid chamber at a temperature of 4 °C for 16 hours and then washed (3 times for 3 minutes each time) using *Phosphate buffered* saline and Tween 20 at 0.005 % (PBS/T). The wells were blocked with 100 μL of human albumin solution at 3 %, diluted in PBS buffer, and then the plate was incubated in a humid chamber at 37 °C for one hour. After this time, the plates were washed (3 times for 3 minutes each time) with PBS/T solution. In each well were added 100 μL of hyperimmune sera from mice immunized with JH1, JH2, JH3, JH4, JH5, JH6, and CQ diluted in PBS (1:100 and 1:200). The plates were incubated in a humid chamber at 37 °C for one hour. Subsequently, the plates were washed (3 times for 3 minutes) with PBS/T solution. 100 μL of the conjugate rabbit anti-mouse polyvalent immunoglobulins (IgG, IgA, and IgM) conjugated to the peroxidase enzyme (HRP-peroxidase Horseradish, Sigma) in 1:1000 dilutions in PBS was added in each well. Again, the plates were incubated in a humid chamber at 37 °C for 1 hour. Then, the plates were washed quickly (three times for 3 min each time) with PBS/T solution. Finally, for the revelation of the chromogenic enzyme reaction, 100 μL of the chromogen ABTS (2,2'-azino-bis(3-ethylbenzthiazoline-6-sulfuric acid, Sigma) and 0.05 % of H_2_O_2_ were added in each well. Then, plates remained in the dark for 15 minutes at room temperature. The reaction was read at 405 nm, at 15 and 30 minutes after the addition of the substrate. Each plate sera from mice BALB/c healthy and PBS were included as a negative control for the assay.

### Assay of cellular stimulation to B lymphocytes from immunized mice

The compounds with some antimalarial activities were proved by an assay of cellular proliferation using MTT (MTT-based reagent; CellTiter 96® Aqueous. One Solution Cell Proliferation Assay from Promega Company). This colorimetric method is based on the reduction of [3-(4,5-dimethylthiazol-2-yl)-5-(3-carboxymethoxyphenyl)-2-(4-sulfophenyl)-2H-tetrazolium, inner salt] to formazan by active cells [23]. The cells used are B lymphocytes from the spleens of mice immunized with compounds like bis-THTT.

### Cytotoxicity of the bis-THTT derivatives compounds

A colorimetric method was used to determine the cytotoxicity of the compounds by using CellTiter 96^®^ AQueous One Solution Cell Proliferation Assay (MTT) from Promega company and cells without any compound were a positive control. 10^5^ SH-SY5Y human neuroblastoma cells were obtained from culture plates using the trypsinization process [24] and the compounds were added to a 96-well culture plate with MEM (Minimum Essential Medium Eagle, Sigma) supplemented and two compounds (JH1, JH2, JH3, JH4, JH5, JH6 and CQ). The plate was incubated at 37 °C for 72 hours in a humidified 5 % CO2 atmosphere. After incubation, 20 μl of MTT were added to the culture wells and the absorbance at 490 nm was measured at different times (0, 1 and 2 h) using a 96-well plate reader [23]. 10^5^ SH-SY5Y human neuroblastoma cells were the proliferation control without any compound and the reduction in absorbance at 490 nm for treated and untreated control cultures was measured and compared at 2 hours.

### Statistical analysis

Each experimental value is presented as the mean of three replicates ± standard deviation. Data normality was verified using the Anderson-Darling test, and homoscedasticity was verified using the Levene test. We performed a comparison of means, using a one-way ANOVA test, to determine if there were statistically significant differences in the parasitemia for the different experimental compounds (taking *p* <0.05 as a significant value and a posteriori Tukey test). The program used to process the data was MINITAB version 17.

## Results and discussion

### Chemistry

**Figure 2.**
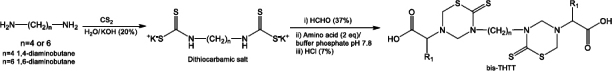
Synthetic route leading to bis-THTT

Alkyl-linked bis-THTTs from series I and II (Figure 1) were synthesized as previously described Coro *et al*. [13,14]. Briefly, one of the commercially available diamines 1,4-diaminobutane or 1,6-diaminobutane reacts with CS_2_ in basic media, allowing for the formation of the expected bis-dithiocarbamate salt intermediate, which undertakes further cyclization in the presence of formaldehyde (HCHO), and the corresponding amino acid in a buffer solution (pH 7.8) (Figure 2). The structural elucidation of the synthesized compounds agreed with the previously reported data [13,15].

### Evaluation of the effect anti-malaria of the experimental compounds in BALB/c mice model with two strains of Plasmodium

**Figure 3.**
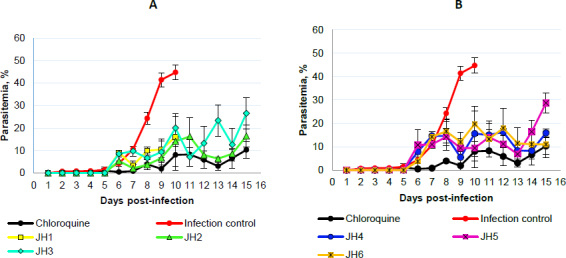
Percentage of parasitemia from mice infected with *P. berghei*. Parasitemia *vs.* days post-infection using female BALB/c mice following the administration of a 4-dose regimen of 20 mg/kg Chloroquine (*n*=3), JH1 (*n*=3), JH2 (*n*=3), JH3 (*n*=3), JH4 (*n*=3), JH5 (*n*=3) and JH6 (*n*=3); administered 72 hours after intraperitoneal (IP) inoculation with 5X10^3^ *P. berghei* parasitized-erythrocytes. Data are shown as total parasitemia (mean percentage of infected red blood cells/total erythrocytes ± SD). Parasitemia was monitored daily by blood smears fixed with absolute methanol and Giemsa stained

**Figure 4.**
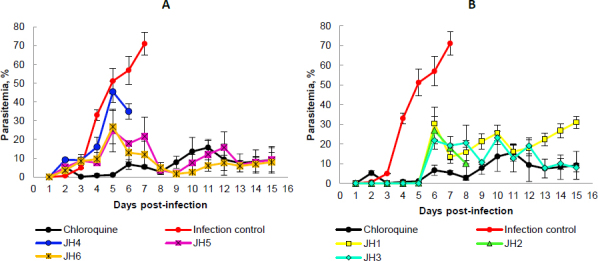
Percentage of parasitemia from mice infected with *P. yoelii*. Parasitemia vs days post-infection using female BALB/c mice following the administration of a 4-dose regimen of 20 mg/kg Chloroquine (*n*=3), JH1 (*n*=3), JH2 (*n*=3), JH3 (*n*=3), JH4 (*n*=3), JH5 (*n*=3) and JH6 (n=3); administered 72 hours after IP inoculation with 5×10^3^ *P. yoelii* parasitized-erythrocytes. Data are shown as total parasitemia (mean percentage of infected red blood cells/total erythrocytes ± SD). Parasitemia was monitored daily by blood smears fixed with absolute methanol and Giemsa stained

After inoculation with 5×10^3^ parasite red blood cell (PRBC) of both strains (*P. berghei* ANKA and *P. yoelii* 17XL), the parasitemia percentage was monitored daily by blood smears and stained with Giemsa’s reagent until day 15 post-infection. In each *Plasmodium* strain infection, the percentage parasitemia was determined as the ratio of PRBCs to the total number of red blood cells. In the following figures, we showed the evaluation *in vivo* of the different bis-THTT compounds and chloroquine (JH1-JH6 and CQ) in the *Plasmodium* infection with both strains. Figure 3 shows the percentage of parasitemia from mice infected with *P. berghei*, and Figure 4 shows the percentage of parasitemia from mice infected with *P. yoelii* strains.

On day five post-infection, we can observe that the percentage parasitemia of infection control increases exponentially until day ten post-infection. All mice infected with *P. berghei* died, showing parasitemia of 44.7 % of the infection control group. On the other hand, for the mice treated with CQ, which corresponds to the positive control of malaria cure, the parasitemia did not exceed 11 %.

The mice evaluated with JH1 ten days post-infection died with an average of 16 % of parasitemia. If we compare this group with the mice of the infection control group, which died the same day but with parasitemia of 44.7 %, we can suggest that the mice inoculated with JH1 died because the compound does not have an antimalarial effect on the parasitic infection; therefore, compound JH1 is not considered effective.

In those mice groups treated with JH2 and JH4, parasitemia did not exceed 16.5 % until day 15 post-infection, suggesting that these compounds could have partial antimalarial activity. Nevertheless, JH2 and JH4 are less efficient than CQ because this treatment presented 9.8 % of parasitemia on day 15 post-infection. In addition, the percentage of parasitemia of JH3 and JH5 fluctuated consistently between 10 and 30 %, suggesting that these bis-THTTs did not control the infection. All mice died on day 15 post-infection with 26.5 and 28.7 % parasitemia for JH3 and JH5, respectively.

Finally, if we look at the percentage of parasitemia for JH6 (11 %) and CQ (9.8 %), we can see that both curves are similar on day 15 post-infection (Fig. 3B). However, parasitemia from day 7 to day 13 post-infection constantly fluctuated between 10 and 20 %, suggesting that compound JH6 did not completely control the infection.

The species *P. berghei* was more sensitive to the compounds JH2, JH4, and JH6. On day 15 post-infection, no significant differences were observed in the percentage of parasitemia between the compounds JH2 (16.45 %), JH4 (10.85 %), JH6 (15.91 %), and chloroquine (10.29 %) (*p* >0.05), demonstrating the efficiency of the treatments. However, the survival percentage was 100 % for mice exposed to chloroquine, decreasing to 66 % for JH2 and JH4 and 33 % for JH6.

In summary, some bis-THTT derivatives compounds evaluated *in vivo* show an antimalarial effect similar to the chloroquine treatment; however, none of them exceeded the chloroquine effect. The two compounds with activity similar to CQ were JH2 (Figure 3A, green line) and JH4 (Fig. 3B, blue line) for the groups of mice infected with *P. berghei*; further, the antimalarial effect of the compounds JH5 and JH6 were lower than CQ regarding survival for *P. yoelii*. In other words, for mice infected with *P. berghei*, the compounds that have control over the infection are JH2 and JH4; and for mice infected with *P. yoelii*, the compounds are JH5 and JH6. These compounds show lower survival than the control group of CQ, suggesting a low efficacy of bis-THTT derivatives compounds as antimalarial drugs compared to CQ.

Concerning *P. yoelii*, there is an evident increase in the percentage of parasitemia of mice treated with JH1, JH2, and JH3 from day five post-infection but with fluctuations up to day 13. From day 13 post-infection, the groups treated with JH2 and JH3 decreased the percentage of parasitemia equal to the group treated with CQ, reaching 10 % for groups JH2 and CQ. For compound JH1, their parasitemia increases up to 30 % on day 15 post-infection (Figure 4A).

Mice treated with JH4, JH5, and JH6 increase their parasitemia from day two to day seven post-infection. The group JH4 reached a maximum parasitemia of 46 % on day five, and on day six, all mice died. This behavior parasitemia curve trend suggests a low efficiency in infection control for *P. yoelii* with this compound (Figure 4B).

For compounds JH5 and JH6, maximum parasitemia was 23 and 27 %, respectively, on day five post-infection. However, for these last two groups, there was a decrease in parasitemia on day eight post-infection, reaching 5 % parasitemia as the group was treated with CQ. The percentage of parasitemia from day nine to day 15 post-infection was very similar for groups JH5, JH6, and CQ, presenting an average of parasitemia of 8.5 % for all these groups. Nevertheless, only one mouse from the group of mice treated with compound JH6 survived, suggesting no effective effect of the compound on parasites. On the other hand, only one mouse died in the group of mice treated with compound JH5. However, in the group of mice treated with CQ, the survival was 100 %.

**Figure 5.**
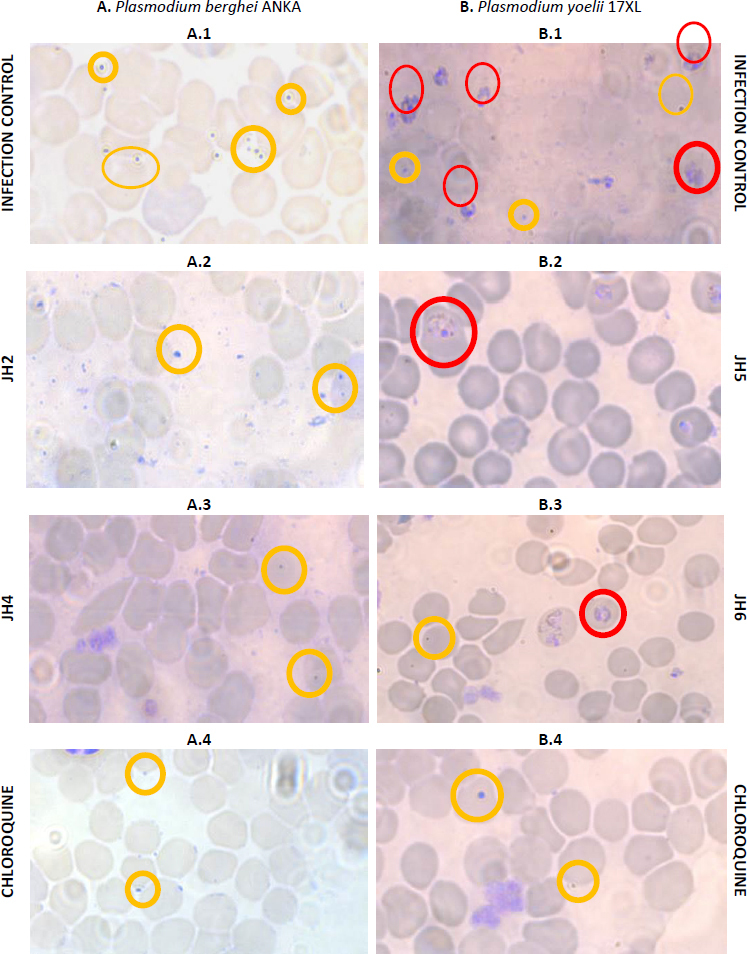
Blood smear of BALB/c mice infected with A: *P. berghei* and B: *P. yoelii*, treated with JH2, JH4, JH5, JH6, and Chloroquine as cure control. The yellow circles show red blood cells with parasites in the merozoite stage, and the red circles point out red blood cells with parasites in the mature schizonte stage

The percentage of parasitemia on day seven post-infection was studied (Figure 5). Blood smears stained with Giemsa’s reagent were used for mice infected with *P. berghei* and with *P. yoelii* with the compounds JH2, JH4, JH5, and JH6, which presented some antimalarial activity. The blood smears of mice infected with *P. berghei* for JH2 (Fig. 5A.2) and JH4 (Figure 5A.3) indicate that there are parasites in the merozoite and trophozoite stage similar to CQ control (Figure 5A.4). Also, for the same day post-infection, in the blood smear corresponding to infection control (Figure 5A.1), more parasitized red blood cells are compared to the treatments with the compounds derived from bis-THTT, and CQ analyzed, in merozoite and trophozoite stages, suggesting that JH2 and JH4 control infection.

On the other hand, the blood smears of mice infected with *P. yoelii* and treated with JH5 (Figure 5B.2) and JH6 (Figure 5B.3) show a similar amount of parasitized red blood cells, which also are comparable to the cure control CQ (Fig. 5B.4). These results indicates that JH5 and JH6 compounds have control over the *P. yoelii* infection.

### Evaluation of the experimental compounds’ immunogenicity and assay of cellular stimulation to B lymphocytes

**Figure 6.**
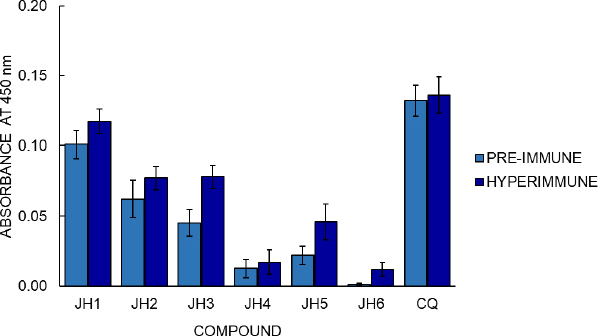
Results of indirect ELISA test, with 10 ug/ml concentration of soluble compounds as antigen. The X-axis represents the 1:200 dilutions of the hyperimmune sera vs. the absorbance on the Y-axis. Each bar represents the immunogenicity of the six experimental compounds: JH1, JH2, JH3, JH4, JH5, JH6, and chloroquine. Additionally, the standard error for each compound is represented on the bars

BALB/c mice were inoculated with compounds JH1 to JH6 to determine the immunogenicity of the bis-THTT derivatives. Later, their pre-immune and hyperimmune sera were used to determine humoral response by indirect ELISA test, using each experimental compound as antigen. The optimal conditions established to evaluate each experimental compound’s humoral response in BALB/c mice by an indirect ELISA were 10 g/ml of antigen concentration and dilution of 1:200 of the serum, determined in previous experiments. The optical density (OD) obtained by indirect ELISA for hyperimmune sera was 0.012, 0.017, 0.046, 0.077, 0.078, and 0.117 to JH6, JH4, JH5, JH2, JH3, and JH1, respectively (Figure 6).

**Figure 7.**
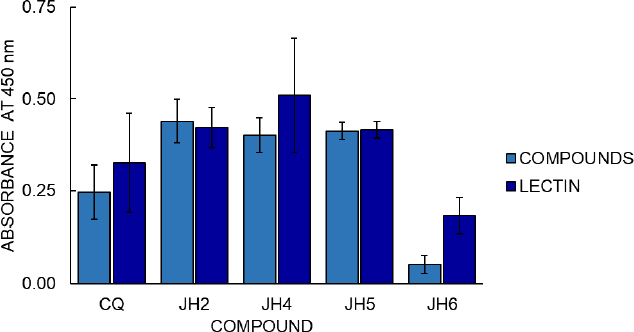
Results of assay of cellular stimulation to B lymphocytes from immunized mice. On the X-axis are represented the treatments obtained as results of cell proliferation to each compound evaluated and the lectin phytohemagglutinin (PHA), as cell proliferation control, in a concentration of 20 μg to all. The absorbance is on the Y-axis. Each bar represents cell proliferation at 2 hours of reading. Additionally, the standard error for each compound is represented on the bars

Cell proliferation assays with the MTT colorimetric method from Promega Company were also carried out (Figure 7) to continue the compounds’ analysis. The cell proliferation assay results with the MTT colorimetric method consider two hours to be the optimal time for reading to determine stimulation to B lymphocytes from immunized mice. Compounds JH2 and JH5 (with OD of 0.439 and 0.412, respectively) show similar or higher cellular stimulation concerning the Phytohemagglutinin (PHA) control of cell stimulation (OD 0.421 and 0.416).

Regarding cellular stimulation to B lymphocytes, the JH1 to JH6 compounds show some antibody response compared to pre-immune sera, suggesting that bis-THH derivatives compounds present immunogenicity when used as antigens. The absorbance value of the hyperimmune sera was greater than the pre-immune sera value, meaning a humoral response variable between the compounds. Considering that CQ is the cure control of malaria and the hyperimmune sera of compounds showed control of malaria infection, we could suggest that the humoral response and antimalarial activity of the bis-THTTs are independent processes.

The cell proliferation assay shows that the most significant amount of metabolically active cells is those in which the mice immunized with JH2 and JH5 obtained high absorbance, in comparison with the compounds JH4, JH6, and CQ, considering that the value of absorbance is directly proportional to the production of formazan as indicative of cell proliferation. On the other hand, these suggest that the absorbance value is directly related to B lymphocyte stimulation, and therefore, the compounds JH2 and JH5 are immunogenic.

The correlation of these results with those obtained in the indirect ELISA showed that these compounds presented absorbances 1.6 and 2 times higher than their pre-immune sera, respectively. Even if we compare JH2 and JH5 with the parasites’ infection control, these compounds control parasitemia for the strains *P. berghei* and *P. yoelii*, respectively. This result indicates that these two bis-THTT have a certain antimalarial action and can stimulate the humoral response.

### Evaluation of cytotoxicity of the bis-THTT derivatives compounds

**Figure 8.**
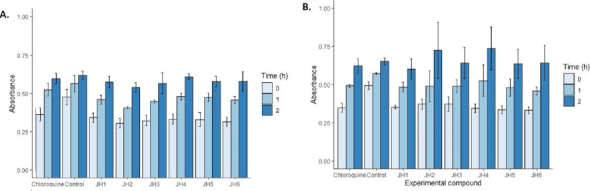
Compounds cytotoxicity determination using a proliferation assay with SH-SY5Y human neuroblastoma cell line (MTT, Promega). (A) Assay with compounds in 10 μg/mL (B) Assay with compounds in 5 μg/mL. The bars represent the OD obtained for the compounds in 0, 1 and 2 hours

In Figure 8, we showed the cytotoxicity effects of the compounds evaluated in cell cultures with SH-SY5Y human neuroblastoma cell line. At one hour, the most toxic compound was JH2 at a concentration of 10 μg/ml with an absorbance of 0.46 OD followed by JH1, JH3, JH5, and JH6 compared to control 0.61 OD according to one-way ANOVA and Tukey’s test (*F* = 7.40; *p* = 0.000). In contrast, JH4 and chloroquine were not toxic. However, at two hours, using 5 μg/ml, all the compounds were not as toxic as control (*p* >0.05).

With respect to the evaluation of cytotoxicity of the bis-THTT derivatives compounds, the MTT colorimetric method for determining cytotoxicity recommends its application for up to 3 hours. However, in the analysis of the compounds with SH-SY5Y human neuroblastoma cell line, it was found that after two hours, the toxicity of the compounds did not show differences compared to the control (*p* > 0.05).

### Preliminary overview of structure-activity relationships (SAR)

**Figure 9.**
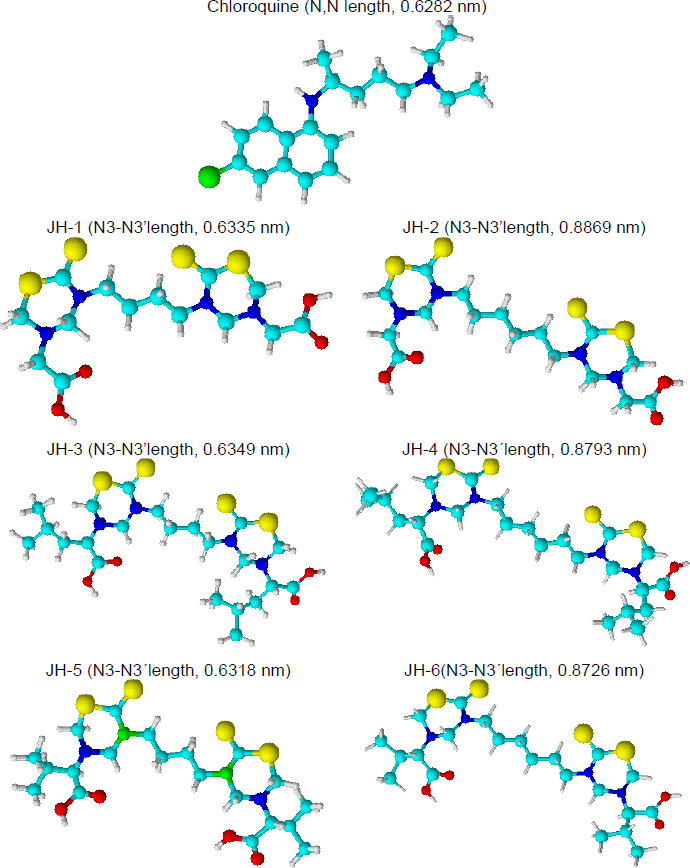
Preliminary three-dimensional structural optimization for chloroquine and bis-THTT derivatives (JH1-JH6) using Chem3D software

The SAR study allows us to tie together chemical or three-dimensional molecular structures and their biological activity. Although the present antimalarial study is introductory and only six bis-THTT derivatives have been analyzed, some SAR conclusions might be established, boosting future works. Basic Chem3D optimization for all structures was carried out to facilitate structural analysis (Figure 9).

Our previous results showed an antimalarial effect for the compounds JH2 and JH4 when the infection with *P. berghei* was carried out. Both compounds have an aliphatic chain of six carbons linked to the two N,N heterocycles, and glycine and leucine (neutral aminoacids) as substituents at N-5 position. On the other hand, compounds JH5 and JH6 showed antimalarial activity for the infection with *P. yoelii*. In this case, the most active structures have different carbon chains linked to N,N-heterocycles but the same substituent (valine residue) at the N-5 position.

The analysis of optimized structures showed that JH1, JH3, and JH5 have N3-N3’ length close to the distance between the nitrogenous atoms of chloroquine, except JH5 in front of *P. yoelii* infection, the other two compounds did not show effective antimalarial activity. For infection by *P. berghei*, the carbon chain and the distance between both heterocycles present in the bis-THTT structures seem to be important. JH2 and JH4 showed higher N3-N3’ lengths in comparison with the chloroquine. On the contrary, to *P. yoelii* infection, the carbon chain length was not significant. Better results were obtained using valine (a neutral and nonpolar amino acid) as a substituent at the N-5 position. Future studies should focus on these preliminary results in the search for structures with better antimalarial activity.

## Conclusions

The first *in vivo* evaluation of bis-THTTs (JH1 to JH6) in a model of BALB/c mice infected with *P. berghei* and *P. yoelii* allowed us to conclude that some of them have an antimalarial effect, which depends on the *Plasmodium* strain used for its evaluation as an antimalarial drug. JH2 and JH4 had an antimalarial effect in the infection with *P. berghei*, while JH5 and JH6 were for infection with *P. yoelii*. However, none of the tested compounds exceeded the efficiency of CQ. The present work demonstrated the *in vivo* evaluation of bis-THTT in a murine model for the first time, obtaining similar biological behavior in the infectious control parasite to chloroquine.

Related to the evaluation of the humoral or antibody-mediated response in mice inoculated with the bis-THTT derivatives compounds, the results of the indirect ELISA test suggest that all bis-THTTs have some humoral response in BALB/c mice and also that there is a relationship between the immunogenicity of the compounds JH2 and JH5 with their antimalarial activity.

In the stimulation of B lymphocytes from spleens of experimental mice with JH2 and JH5 as antigens, the MTT colorimetric proliferation kit and similar cell proliferation activity to PHA mitogen as proliferation control was observed. This result suggests that bis-THTT appears to be able to stimulate the humoral response, and the best stimulation of B lymphocytes was JH2.

Preliminary structure-activity relationship analysis of the chloroquine (CQ) and the bis-THTT (JH1 to JH6) suggested that antimalarial activity would be related to the distance of alkyl-linked the N3 of each thiadiazine ring and the amino acid residue nature at N5 position. The better results against *P. berghei* were obtained for JH2 and JH4, which have an alkyl chain of six carbons and neutral amino acid residues such as glycine or leucine in position five of the heterocycles. Related to antimalarial activity in mice infected for *P. yoelii*, JH5 and JH6 showed better results, evidencing the structural importance of the valine residue at N5 and not the distance between heterocycles.
